# Genome characterization of a Korean isolate of porcine epidemic diarrhea virus

**DOI:** 10.1128/mra.00118-23

**Published:** 2023-12-20

**Authors:** Dae-Min Kim, Sung-Hyun Moon, Seung-Chai Kim, Taek Geun Lee, Ho-Seong Cho, Dongseob Tark

**Affiliations:** 1 Laboratory for Infectious Disease Prevention, Korea Zoonosis Research Institute, Jeonbuk National University, Iksan, South Korea; 2 College of Veterinary Medicine, Jeonbuk National University, Iksan, South Korea; Katholieke Universiteit Leuven, Leuven, Belgium

**Keywords:** porcine epidemic diarrhea virus, genome, Korean, pathogenicity

## Abstract

Porcine epidemic diarrhea (PED) outbreaks occur annually in the Republic of Korea. The complete genome sequence of the PED virus isolate CKK1-1 obtained from an infected pig was determined. The genome is 28,037 nt long, excluding the poly(A) tail, and contains seven open reading frames flanked by two untranslated regions.

## ANNOUNCEMENT

Porcine epidemic diarrhea virus (PEDV) is highly contagious and causes enteric swine disease (1, 2). PEDV is an enveloped, single-stranded, positive-sense RNA virus that belongs to the family *Coronaviridae*, order *Nidovirales*. Based on genetic analysis, PEDV strains are mainly divided into two groups—genogroup 1 constituting classical or recombinant and low-pathogenic strains and genogroup 2 constituting field epidemic or pandemic and high-pathogenic strains (3). In November 2019, a PED outbreak occurred in Pocheon, Gyeonggi Province, Republic of Korea. Intestinal samples, which were originated from the area, were collected from pig case submissions to the Jeonbuk National University Veterinary Diagnostic Center (JBNU-VDC) to detect PEDV. This study has been approved and conducted under the Institutional Animal Care and Committee of Jeonbuk National University (IACUC, protocol #: JBNU2021-078) (4, 5). For next-generation sequencing (NGS), viral RNA from the intestines was extracted using the QIAamp Viral RNA Mini Kit (Qiagen, Germany) following the manufacturer’s instructions.

Reverse transcription and library preparation were performed using cDNA Synthesis and Library Preparation kits (Celemics, Republic of Korea). Subsequently, the sequencing library and capture probes, which were designed and chemically synthesized, were hybridized to capture the regions of the PEDV library using the Celemics Target Enrichment Kit (Celemics). The captured fragments were amplified to enrich the number of PEDV specific reads using PCR-based approaches according to the manufacturer’s instructions. The enriched library was sequenced using an Illumina MiSeq system (2 × 150 bp; Illumina, USA). The raw reads were assembled *de novo,* and quality control of the reads was conducted using CLC Genomics Workbench (version 8.5.1; CLC Bio, Qiagen, USA). In total, 4,285,176 reads were generated; the number of reads assembled into the PEDV genome was 788,065 and the coverage depth was 2,899.33×.

**Fig 1:**
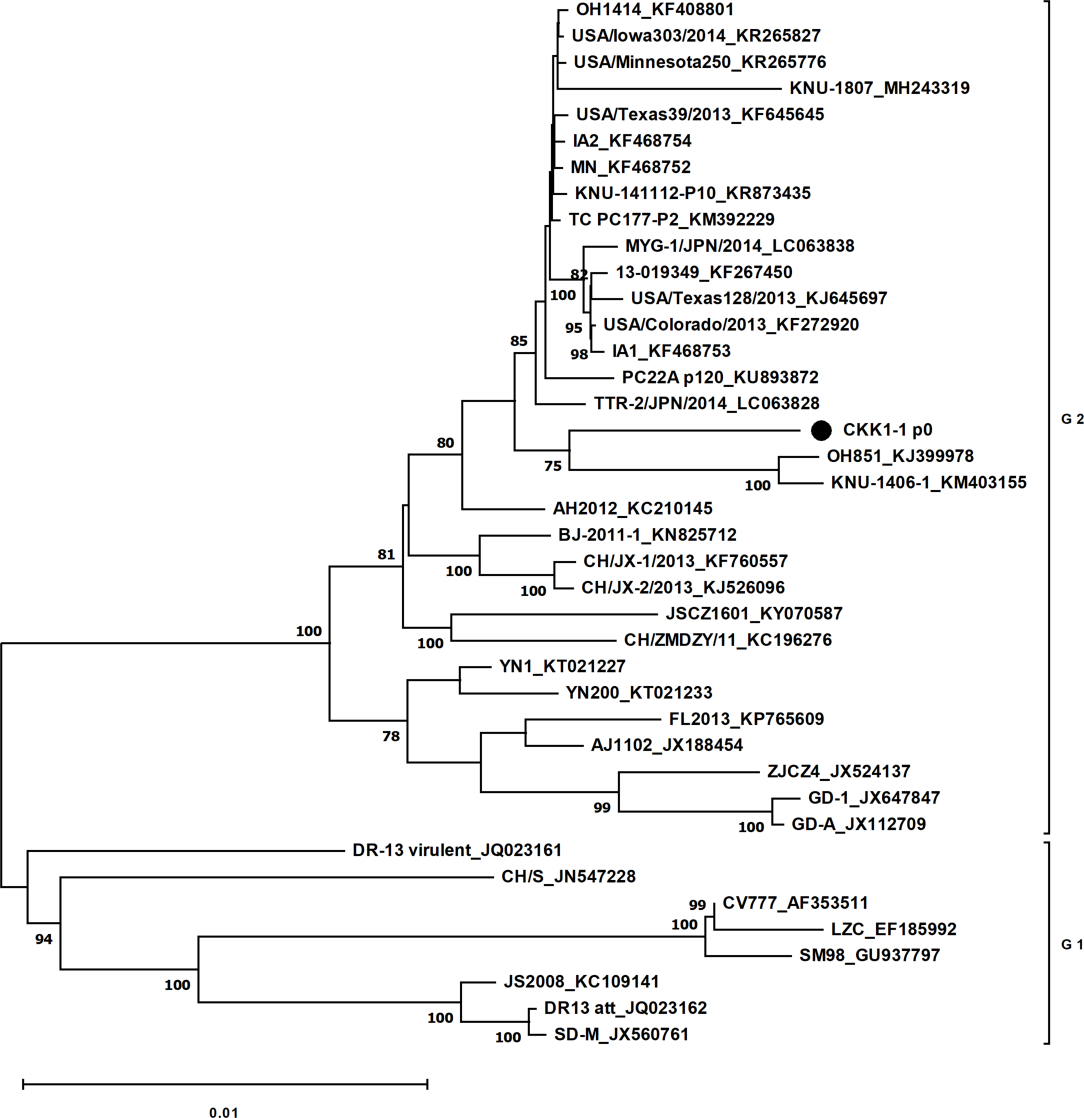
Phylogenetic analysis of CKK1-1 strain based on the nucleotide sequence of whole genome. Phylogenetic tree of the whole genome. The tree was generated via the neighbor-joining method using the MEGA X software. The model of genetic evolutionary distance was computed using the Tamura-Nei method, and the percentage of reliability values for each node was determined via bootstrap analysis with 1,000 replicates. Bootstrap percentages ≥ 70% are shown at the nodes. Scale bar indicates nucleotide substitutions per site.

The strain was designated CKK1-1. Its complete genome sequence was 28,037 nt long, excluding the 3′-end poly(A) tail, and had a G + C content of 41.67%. To examine open reading frames (ORFs), the genome was compared with that of CV777 strain (accession no. AF353511) using Snapgene viewer (version 6.1.0; USA). Like other isolates of PEDV, CKK1-1 has seven ORFs. ORF1a (nt 292–12,645) and ORF1b (nt 12,810–20,636) encode large nonstructural proteins, whereas the remaining ORFs encode four structural proteins—the spike protein (encoded by nt 20,633–24,793); a hypothetical protein encoded by ORF3 (nt 24,793–25,467); the envelope protein (nt 25,448–25,678); the membrane protein (nt 25,686–26,366); and the nucleocapsid (nt 26,378–27,703) (6). The coding sequences are flanked by a 5′ untranslated region (UTR) (nt 1–291) and a 3′ UTR (nt 27,704–28,037). To elucidate the genetic relationship between CKK1-1 and other PEDV isolates, a multiple sequence alignment of 39 PEDV sequences obtained from GenBank was performed using Clustal W. A phylogenetic tree was constructed using MEGA X (5). At the whole-genome level, CKK1-1 belongs to genogroup-2 and is closely clustered with the OH851 isolate in an adjacent clade (Fig. 1) (7). These data provide valuable insights into the epidemiology and evolution of circulating PEDV strains in the Republic of Korea.

## Data Availability

The genome information and raw sequencing reads of PEDV isolate CKK1-1 were deposited in GenBank and BioProject under accession numbers OM714830 and PRJNA935479, respectively.
